# Perceiving Object Shape from Specular Highlight Deformation, Boundary Contour Deformation, and Active Haptic Manipulation

**DOI:** 10.1371/journal.pone.0149058

**Published:** 2016-02-10

**Authors:** J. Farley Norman, Flip Phillips, Jacob R. Cheeseman, Kelsey E. Thomason, Cecilia Ronning, Kriti Behari, Kayla Kleinman, Autum B. Calloway, Davora Lamirande

**Affiliations:** 1 Department of Psychological Sciences, Ogden College of Science and Engineering, Western Kentucky University, Bowling Green, Kentucky, United States of America; 2 Department of Psychology & Neuroscience Program, Skidmore College, Saratoga Springs, New York, United States of America; University of Bath, UNITED KINGDOM

## Abstract

It is well known that motion facilitates the visual perception of solid object shape, particularly when surface texture or other identifiable features (e.g., corners) are present. Conventional models of structure-from-motion require the presence of texture or identifiable object features in order to recover 3-D structure. Is the facilitation in 3-D shape perception similar in magnitude when surface texture is absent? On any given trial in the current experiments, participants were presented with a single randomly-selected solid object (bell pepper or randomly-shaped “glaven”) for 12 seconds and were required to indicate which of 12 (for bell peppers) or 8 (for glavens) simultaneously visible objects possessed the same shape. The initial single object’s shape was defined either by boundary contours alone (i.e., presented as a silhouette), specular highlights alone, specular highlights combined with boundary contours, or texture. In addition, there was a haptic condition: in this condition, the participants haptically explored with both hands (but could not see) the initial single object for 12 seconds; they then performed the same shape-matching task used in the visual conditions. For both the visual and haptic conditions, motion (rotation in depth or active object manipulation) was present in half of the trials and was not present for the remaining trials. The effect of motion was quantitatively similar for all of the visual and haptic conditions–e.g., the participants’ performance in Experiment 1 was 93.5 percent higher in the motion or active haptic manipulation conditions (when compared to the static conditions). The current results demonstrate that deforming specular highlights or boundary contours facilitate 3-D shape perception as much as the motion of objects that possess texture. The current results also indicate that the improvement with motion that occurs for haptics is similar in magnitude to that which occurs for vision.

## Introduction

One hundred and seventy-eight years ago [1] Charles Wheatstone expressed his view that relative motion between environmental objects and human observers produced as good a perception of solid object shape as that that occurs during binocular stereopsis. In particular, Wheatstone (p. 380) said:

“when different projections of the same object are successively presented… the form to which they belong is completely characterized”.

Later in the nineteenth-century, Hermann Helmholtz [2] expressed a similar belief (p. 297), that motion permits observers to form “correct apperceptions of the material shapes of their surroundings”. Perhaps surprisingly, good empirical evidence to support Wheatstone (and von Helmholtz) was not obtained for more than 100 years; that evidence was published in 1953 by Wallach and O'Connell [3] in a now well-known article entitled “The kinetic depth effect”. Wallach and O'Connell found that when human observers viewed deforming 2-dimensional (2-D) cast shadows of rotating 3-dimensional (3-D) objects, those deforming 2-D shadows appeared to be solid. These perceptions of solid shape occurred whenever the projected images contained motions of trackable object features, such as corners and object vertices; when these trackable object features were absent, however, Wallach and O'Connell found that the projected motion sequences appeared not as rigid rotations of a solid object in depth, but as nonrigid deformation. Later researchers [4–9] extended these investigations of the kinetic depth effect in human observers using computer-generated patterns and motion sequences. In such investigations, the perception of 3-D structure was enabled by the projected motions of trackable features, such as random dots or surface texture.

Over the past 35 years, a parallel series of investigations produced computational models and algorithms that can recover 3-D structure from motion [10–15]. Such models require the presence of identifiable object features, such as surface texture elements or sharp corners. The 2-D projected motions of these identifiable features are used (along with certain assumptions, such as object rigidity, fixed-axis motion, etc.) to recover information about 3-D object shape. Over the past twenty years, however, psychophysical research using human observers has shown that the presence of identifiable surface features is not necessary for the effective perception of solid object shape from motion—for example, observers can discriminate 3-D object shape from optical patterns containing only boundary contours, shading, and/or specular highlights [16–19]. Similarly, the presence of identifiable surface features or texture is not necessary for human observers to effectively perceive local aspects of 3-D object shape, such as the orientation of local surface patches [20–23].

While it has long been known that object motion leads to a significant improvement in the visual perception of 3-D object shape (i.e., the kinetic depth effect), almost no similar research has investigated the haptic (active touch) perception of 3-D object shape. Gibson [24] used a set of six 2-D outline shapes (in actuality, these outline shapes were 1-inch diameter “cookie cutters”, which are normally pressed into rolled dough to create differently-shaped cookies). In one condition (passive touch) a randomly-selected cookie cutter was pressed into the palm of a participant's hand on any particular trial, and the participant was required to identify it. In the active touch condition, the participants were allowed to actively explore the cookie cutters using their hands and fingers—the task was again to identify the single object presented on any given trial. Gibson found that while the performance in the active touch condition was excellent (95 percent correct identification), performance in the passive condition was considerably worse (49 percent correct identification). One can legitimately consider this to be a haptic analog to the visual “kinetic depth effect”: the shape-identification performance obtained with relative motion of the object and hand/fingers was superior to that obtained from the tactile stimulation produced by a stationary object and hand. In a more recent investigation by Pont, Kappers, and Koenderink evaluating tactile curvature discrimination [25], however, performance for dynamic touch was no better than for static touch (see their Figure 17)—in this case, there was no “kinetic depth effect” for the haptic perception of shape.

One purpose of the current set of experiments was to compare the improvement that occurs with motion across vision and haptics—does the introduction of object motion lead to similar quantitative improvements in 3-D shape identification performance for vision and haptics? A direct comparison (using the same set of object shapes) between visual and haptic shape identification and how they are affected by object motion has never been performed. An equally important purpose of the current research was to evaluate whether the magnitude of the kinetic depth effect for the recognition/identification of shape is different when object surfaces contain texture (a situation where traditional computational models can operate and recover 3-D structure) and when they do not (i.e., when moving surfaces are defined only by boundary contours and/or specular highlights; most computational models cannot recover 3-D shape under these conditions).

## Experiment 1

### Methods

#### Apparatus

The experimental stimulus objects in the visual conditions were generated by an Apple MacPro computer (2 quad-core processors) and were displayed on a 22-inch Mitsubishi Diamond Plus 200 color monitor (resolution was 1280 x 1024 pixels). The stimulus rendering was accelerated by an ATI Radeon HD 5770 graphics processor. The viewing distance was 100 cm.

#### Experimental Stimuli

**Fig 1:**
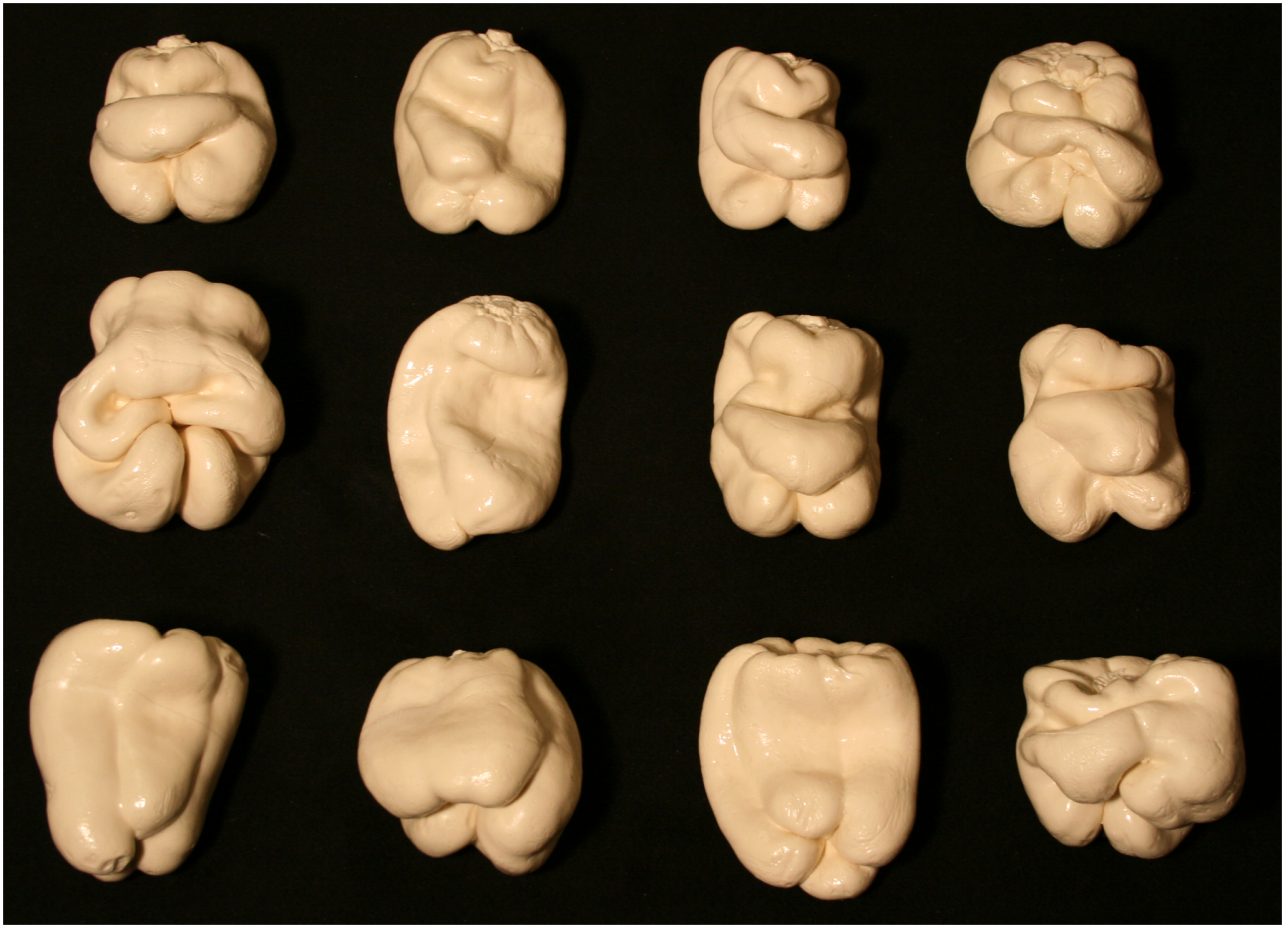
A photograph of the naturally-shaped solid objects (replicas of bell peppers, *Capsicum annuum*) used in Experiment 1. They are arranged in numerical order (1–12) from top-left to bottom right. These objects (and subsets of them) have been used in multiple previous investigations [18, 26–28].

**Fig 2:**
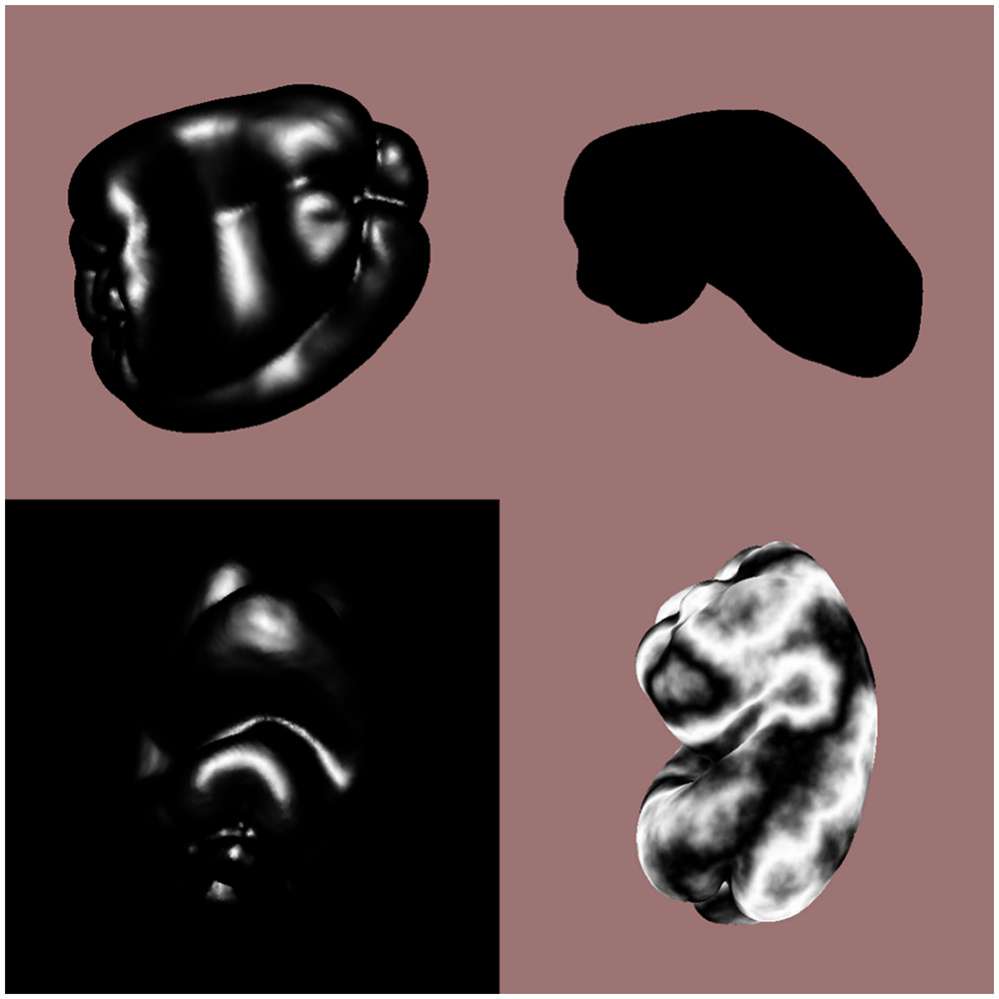
Example stimulus displays used in the visual conditions of Experiment 1. From bottom-left going clockwise are depicted objects defined by 1) specular highlights without occlusion boundary contours, 2) specular highlights with occlusion boundary contours, 3) occlusion boundary contours only (i.e., silhouettes), and 4) volumetric/solid texture, where the surface markings resemble those of marble.

**Fig 3:**
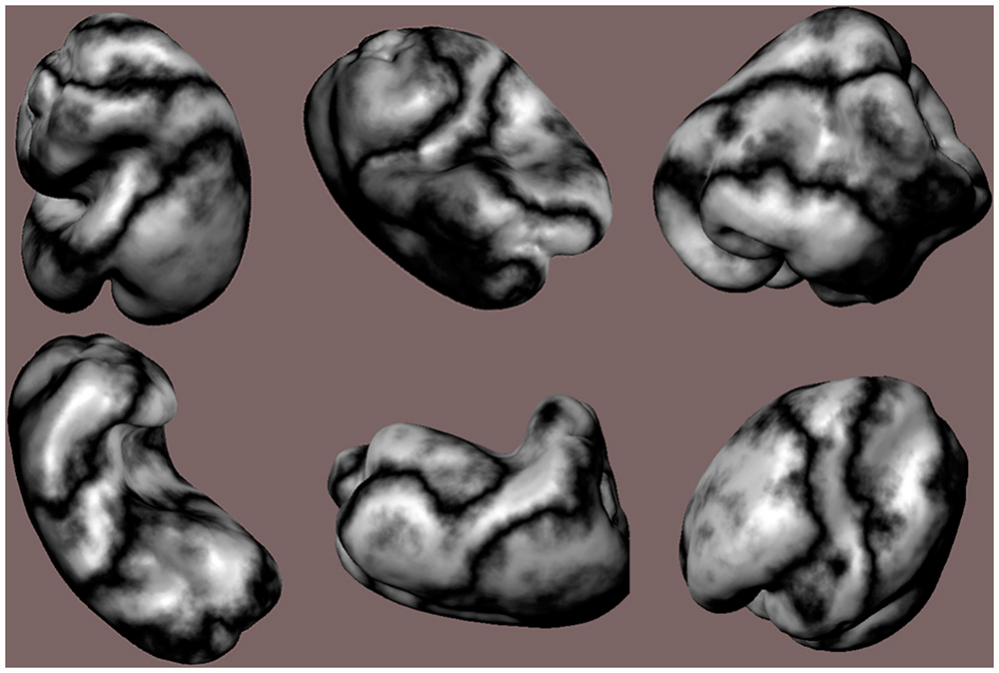
Examples of the solid/volumetric texture applied to six of the 12 stimulus objects: from top-left to bottom-right are depicted bell peppers 1, 2, 5, 6, 9, and 10. To better appreciate the stimulus shapes, the renderings in this figure include image shading and specular highlights as well the results of solid texturing.

The stimulus objects were plastic copies of 12 ordinary bell peppers (*Capsicum annuum*, see Fig 1) that have been used in previous research [26–28]. The solid shapes of the bell pepper replicas were digitally scanned using a 3-D laser scanner (NextEngine, Inc.). The object surfaces in the visual conditions were defined by the positions and orientations of about 74,000 triangular polygons. The objects were optically presented to the participants 1) as silhouettes (i.e., shapes defined only by outer boundary contours), 2) by specular highlights (without boundary contours), 3) by specular highlights combined with boundary contours, and 4) by boundary contours combined with solid/volumetric texture. Fig 2 illustrates these conditions, while Fig 3 demonstrates the results of the solid texturing in more detail.

All rendering was done using the standard OpenGL reflectance model [29]. For the boundary contour conditions all shading coefficients for the ambient, Lambertian diffuse, and specular components (e.g. k_a_, k_d_ & k_s_) were made equal to 0.0, resulting in an all-black silhouette rendered against a background of {r, g, b} = {0.616, 0.455, 0.455}. In the specular highlight conditions the objects were rendered with a specular coefficient k_s_ = 1.0, a highlight color of {r, g, b} = {1.0, 1.0, 1.0} and a shininess exponent of 20.0 [29–30]. For this condition, a single infinite light source was placed such that it illuminated the objects from a direction corresponding to θ_slant_ = 30° and ϕ_tilt_ = 45°. For the boundary contour inclusive highlight condition the objects were displayed against a background of {r, g, b} = {0.616, 0.455, 0.455}. In the highlights only condition, the objects were rendered onto a black background, thus making the boundary invisible. For the texture condition, a solid (i.e., volumetric) texture was created using 3-D value-noise [31–32]. In effect, material was then “carved away” from the solid block of texture so that the solid shape of the stimulus objects was all that remained; this process led to the creation of surface markings that somewhat resemble those of marble. The diffuse and specular (k_d_, k_s_) lighting coefficients were set to 0.0 while the ambient coefficient, k_a_ was set equal to 1.0. This resulted in the rendering of only texture markings with no shading or specular highlights. As in the other boundary contour inclusive conditions, the objects were rendered against a background of {r, g, b} = {0.616, 0.455, 0.455}.

Physical replicas of the bell peppers were used in the haptic condition. Rubber molds (Evergreen 30, Smooth-on, Inc.) were created from the original set of bell peppers; solid copies of the original objects were then produced by pouring liquid plastic (C-1506, Smooth-on, Inc.) into the molds.

#### Procedure

In all conditions, a single randomly-chosen bell pepper was presented on any given trial—the participants' task was to indicate which of 12 simultaneously visible, physical bell peppers (laid out on a table in front of the participants at a viewing distance of 55 cm) possessed the same 3-D shape. No feedback was provided to the participants regarding the accuracy of their shape-matching responses. Each single stimulus object was presented for 12 seconds in a random orientation. In the four visual conditions (surfaces defined by boundary contours only, boundary contours+specular highlights, boundary contours+volumetric texture, specular highlights only), the objects were either presented statically or in motion (rotated in depth about a Cartesian vertical axis located in the plane of the computer monitor; the angular rotation per frame transition was 2 deg, while the frame update rate was 75 Hz). The viewing transform of the computer generated stimuli was chosen to mimic the viewing angle of the individual physical stimuli—approximately 8° of visual angle. In the haptic condition, the objects were either held statically (static enclosure) [33] or were actively manipulated by the participant using both hands [28]. The presentation type (4 visual conditions + 1 haptic condition) was a between-groups manipulation, while the presence or absence of object motion was a within-groups manipulation. Each participant made a total of 60 shape-matching judgments (30 judgments with object motion & 30 judgments without object motion). Half of the participants judged the objects with motion first, while the remaining half first judged objects presented without motion.

#### Participants and ethics

Thirty adults participated in the experiment (mean age was 22.7 years, SD = 3.7; their ages ranged from 20 to 36 years). The participants' vision was assessed with a standard eye chart (Precision Vision catalog number 2195) at a distance of 1 m. All participants possessed normal, or corrected-to-normal visual acuity. The experiment was approved by the Western Kentucky University Institutional Review Board; all participants gave written consent prior to participation in the experiment.

### Results and Discussion

**Fig 4:**
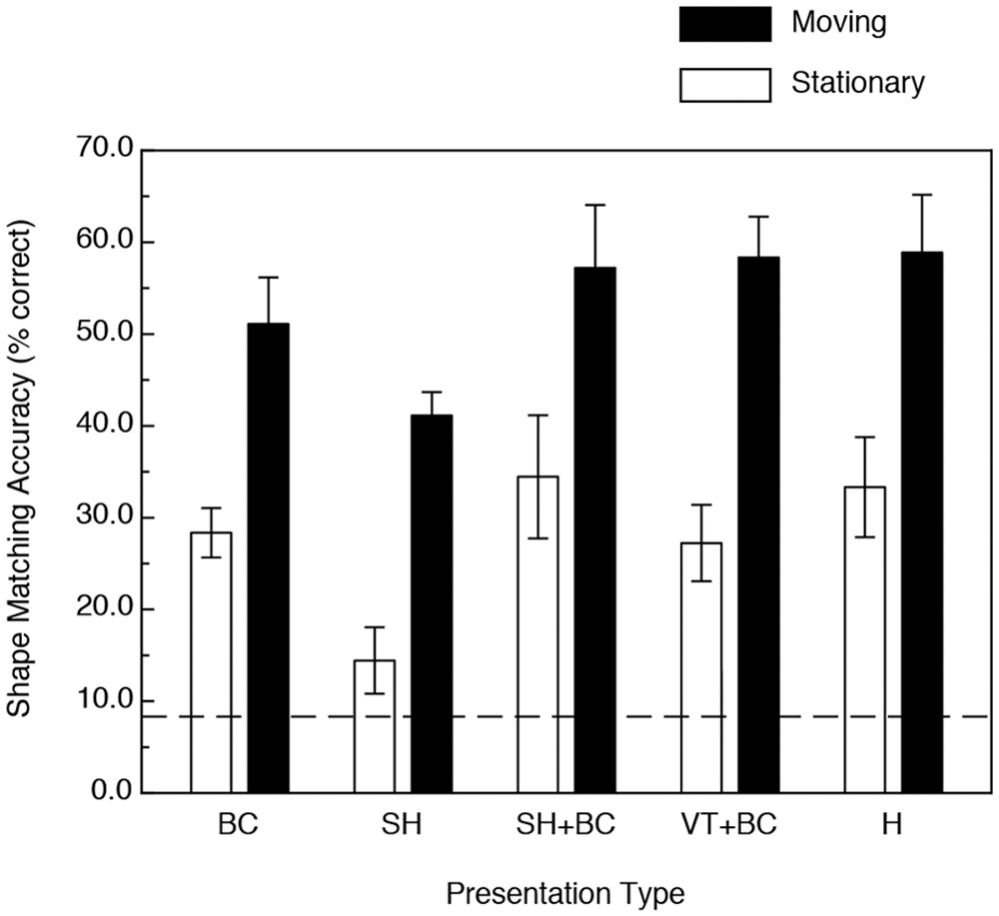
Results of Experiment 1. The participants’ shape-matching accuracies (in terms of percent correct) are plotted as functions of both 1) the stimulus presentation type and 2) the presence or absence of object motion/active haptic manipulation. BC = boundary contours only, SH = specular highlights only, SH+BC = specular highlights with accompanying boundary contours, VT+BC = volumetric texture with accompanying boundary contours, H = haptic manipulation. The error bars indicate ± 1 SE. The dashed line indicates chance performance.

**Fig 5:**
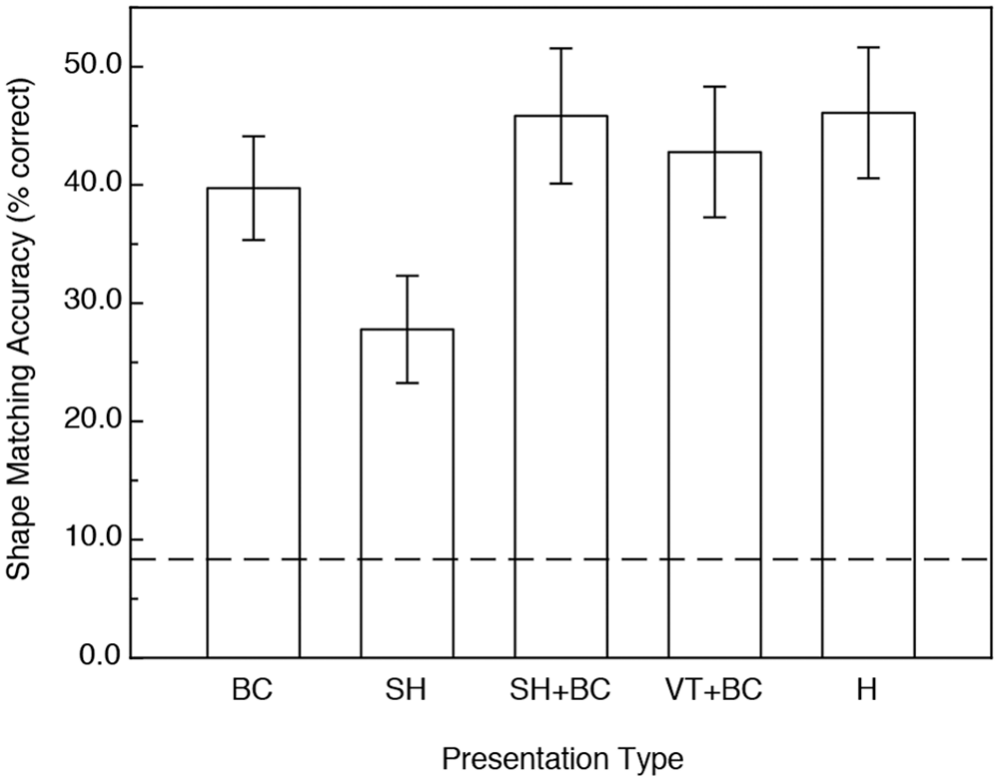
Results of Experiment 1, plotting the participants’ overall shape matching performance for the various stimulus presentation types. BC = boundary contours only, SH = specular highlights only, SH+BC = specular highlights with accompanying boundary contours, VT+BC = volumetric texture with accompanying boundary contours, H = haptic manipulation. The error bars indicate ± 1 SE. The dashed line indicates chance performance.

The participants' results are shown in Fig 4. As is readily evident, the participants’ shape matching performance was much higher (93.5% higher) in the conditions with motion and/or active haptic manipulation (F(1, 25) = 70.8, p < .000001, η^2^_p_ = 0.74). This improvement with motion (relative to performance obtained in the static conditions) is the kinetic depth effect. One can also see from an inspection of Fig 4 that the magnitude of the kinetic depth effect was essentially identical for all of the visual and haptic conditions (i.e., there was no motion x presentation type interaction: F(4, 25) = 0.25, p = .91). There was a significant main effect of presentation type, however (F(4, 25) = 4.2, p < .01, η^2^_p_ = 0.41). As can be seen from Fig 5, the participants' overall shape-matching performance was similar for all of the visual and haptic conditions, except for the visual condition where the objects were defined only by specular highlights (with no accompanying boundary contours). Performance for that condition (specular highlights only) was significantly lower than that obtained for the others according to Fisher LSD (least significant difference) post-hoc tests (all p < .05).

## Experiment 2

In Experiment 1 we found for a particular class of naturally-shaped objects that the presence of motion (whether visual motion or active haptic exploration) produces a large improvement in the ability to perceive shape. The shapes of these particular bell peppers have been well characterized mathematically by Norman, Phillips, et al. [28]. Despite the fact that the 12 individual peppers possess distinctive global shapes and “look different” (Fig 1), the distributions of the objects’ local surface shapes (i.e., shape indices) and surface curvature magnitudes are quite similar (see Fig 2 of Norman, Phillips, et al.). The purpose of Experiment 2 was to extend the current investigation to evaluate the effects of visual and haptic motion for a set of solid objects that were purposefully designed to possess a wide range of stimulus complexities.

### Methods

#### Apparatus

The experimental stimulus objects in the visual conditions were generated and displayed on an Apple iMac computer (27-inch, mid 2011). The monitor was set to a resolution of 1280 x 1024 pixels to match the previous experiment. Stimulus rendering was accelerated by an AMD Radeon HD 6770M graphics processor. The viewing distance was 100 cm.

#### Experimental Stimuli

**Fig 6:**
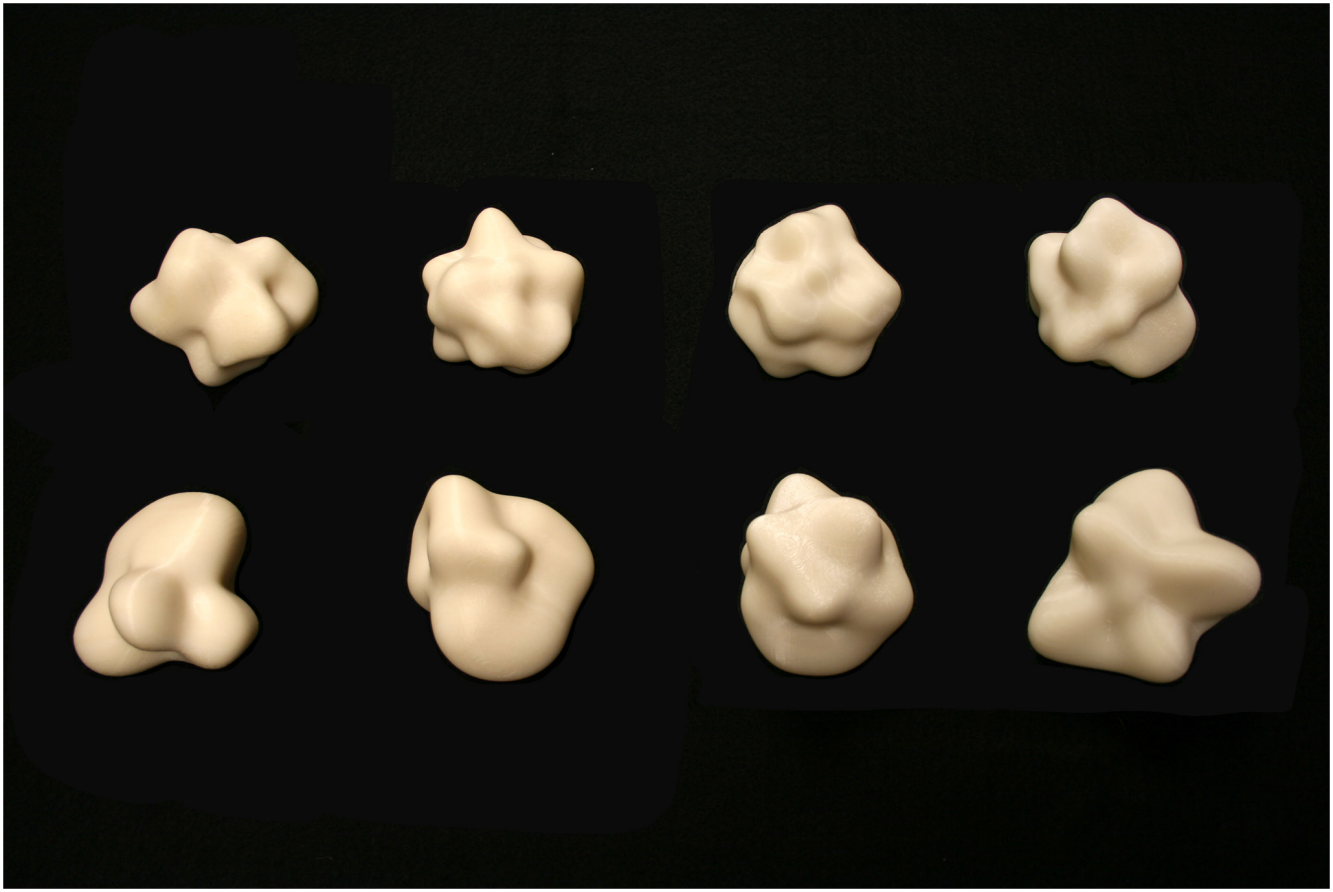
A photograph of the solid objects (glavens) used as stimuli in Experiment 2. The glavens are ordered in terms of stimulus complexity; the complexity increases from the bottom-left to the upper right.

The stimulus objects were 8 pseudo-random objects called ‘glavens’ (see Fig 6) that have been used in previous research [34–37]. The objects were optically presented in the same fashion as in Experiment 1 using the same rendering conditions. Glavens were chosen for this experiment as they can be produced with continuously varying amounts of geometric complexity [34, 36]. The eight stimuli used in this study were selected from a set previously used [36]. When ordered in terms of complexity from simplest to most complex, each one (visually) differs from the next by approximately one jnd. Physical copies of the glavens were created in ABS-M30 plastic using a uPrint SE 3-D printer (Stratasys, Inc.). The objects were printed in ‘ivory’ color and were left unfinished (e.g., without sanding, painting or other surface treatment).

#### Procedure

The procedure was identical to that of Experiment 1. Depending on the condition, the objects were presented visually or haptically for 12 seconds, with and without motion (or active exploration in the haptic condition). The 8 target glavens were visible on the table in front of the participant. Each participant made a total of 60 shape-matching judgments (30 judgments with object motion & 30 judgments without object motion). Half of the participants judged the objects with motion first, while the remaining half first judged objects presented without motion.

#### Participants and ethics

Twenty-seven adults participated in the experiment (mean age was 19.2 years, SD = 1.2 years). The participants' vision was assessed with a standard Snellen-style eye chart (Precision Vision catalog number 5002). All participants possessed normal, or corrected-to-normal visual acuity. The experiment was approved by the Skidmore College Institutional Review Board; all participants gave written consent prior to participation in the experiment.

### Results and Discussion

**Fig 7:**
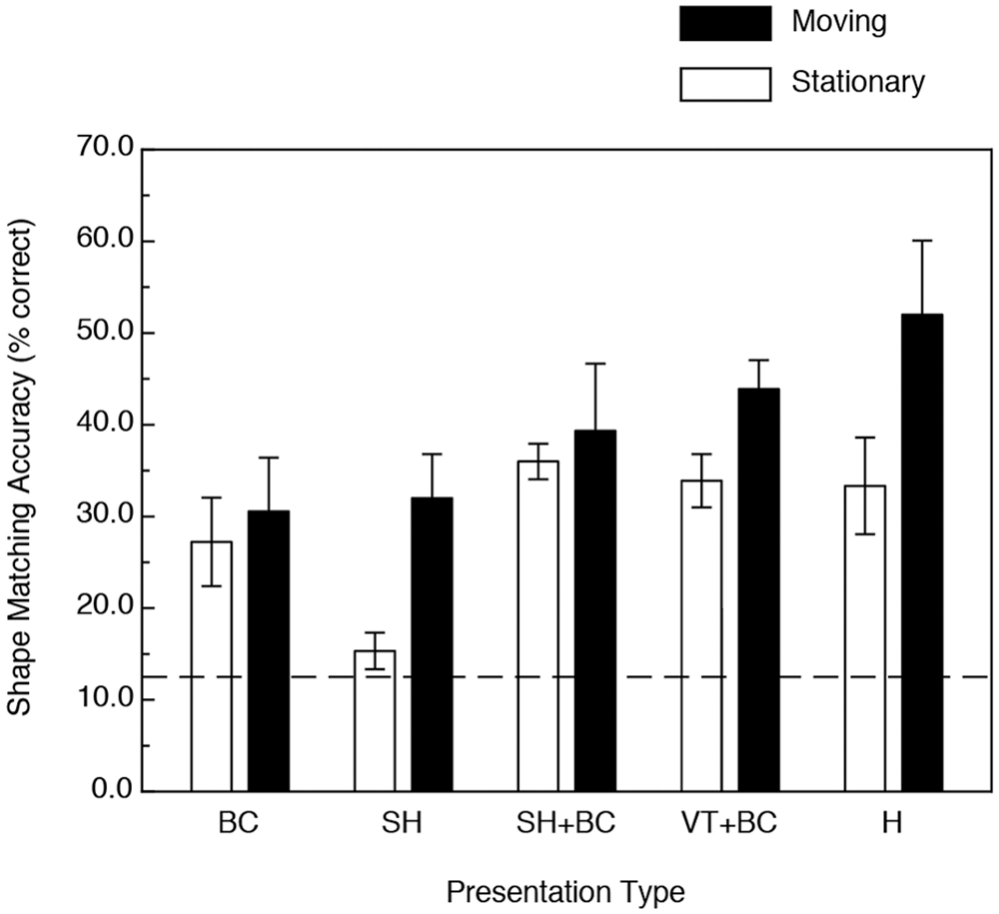
Results of Experiment 2. The participants’ shape-matching accuracies (in terms of percent correct) are plotted as functions of both 1) the stimulus presentation type and 2) the presence or absence of object motion/active haptic manipulation. BC = boundary contours only, SH = specular highlights only, SH+BC = specular highlights with accompanying boundary contours, VT+BC = volumetric texture with accompanying boundary contours, H = haptic manipulation. The error bars indicate ± 1 SE. The dashed line indicates chance performance.

**Fig 8:**
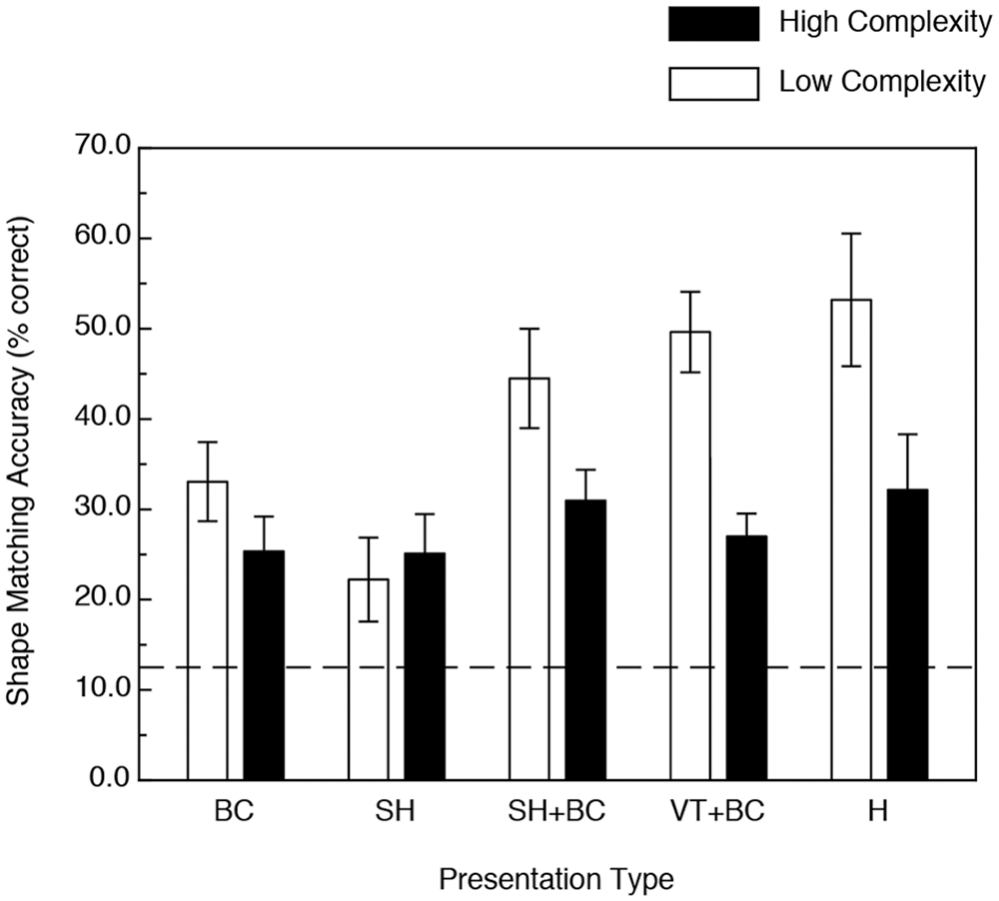
Results of Experiment 2. The participants’ shape-matching accuracies (in terms of percent correct) are plotted as functions of both 1) the stimulus presentation type and 2) stimulus object complexity (the 8 stimulus objects were partitioned into two groups possessing low and high stimulus complexity). BC = boundary contours only, SH = specular highlights only, SH+BC = specular highlights with accompanying boundary contours, VT+BC = volumetric texture with accompanying boundary contours, H = haptic manipulation. The error bars indicate ± 1 SE. The dashed line indicates chance performance.

**Fig 9:**
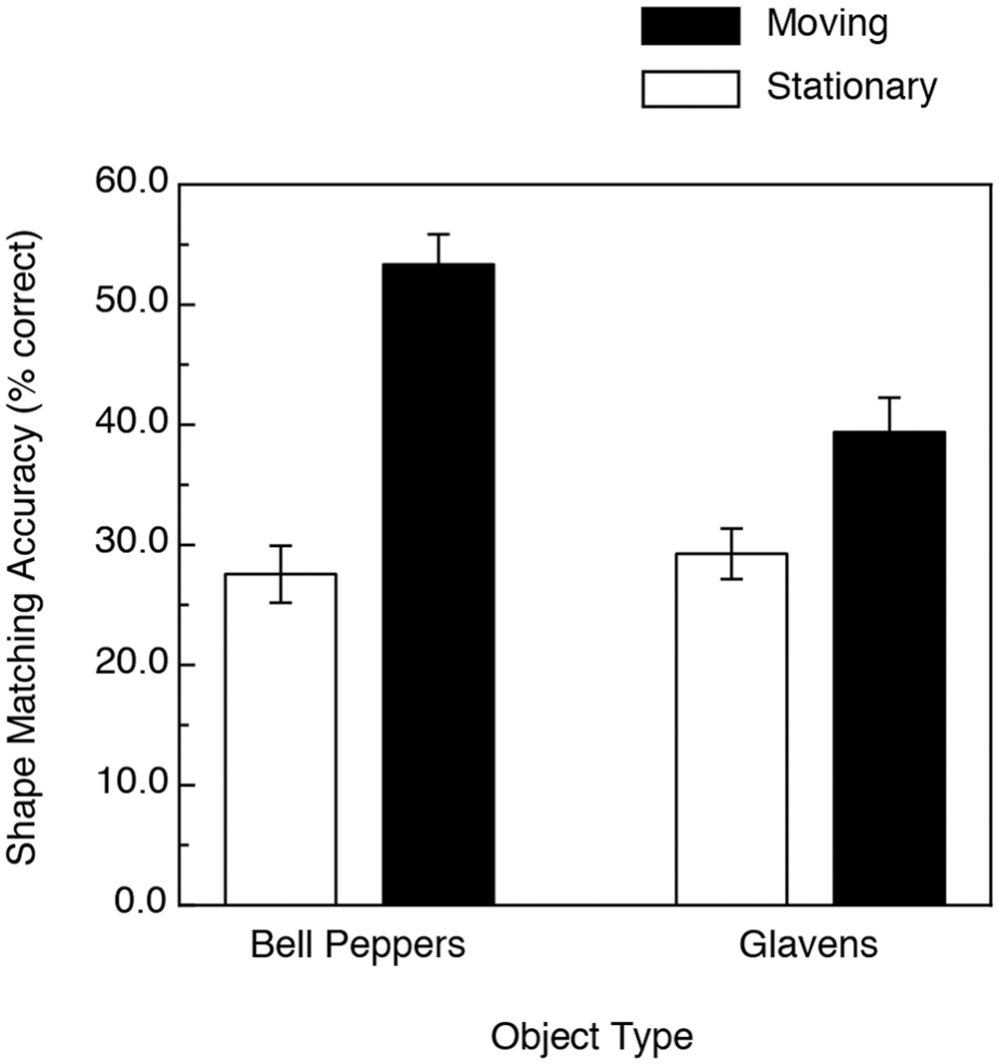
A comparison of the results of Experiments 1 (bell pepper replicas) and 2 (glavens). The error bars indicate ± 1 SE.

The results are shown in Figs 7–9. Fig 7 plots performance as functions of stimulus presentation type and the presence/absence of object motion. As with Experiment 1, the participants’ shape-matching performance was significantly higher (34.6 percent higher overall) in the conditions with motion and/or active haptic manipulation (F(1, 22) = 13.68, p < .001, η^2^_p_ = 0.38). Again, there was no motion x presentation type interaction—F(4, 22) = 1.28, p = .19) and there was a significant main effect due to presentation type—F(4, 22) = 4.0, p < .01, η^2^_p_ = 0.42. Note that this is the same pattern of results found in Experiment 1 with the bell peppers, but with smaller effect sizes throughout.

The stimuli (glavens) used in Experiment 2 are parameterized such that they can be ordered in terms of relative complexity [34, 36]. We repeated our analysis after partitioning the stimulus objects into low- and high-complexity groups. The first four stimuli were placed in the low-complexity group and the last four into the high-complexity group. Fig 8 shows the results of this partitioning. First of all, there was a main effect of stimulus complexity, such that the participants’ performance was significantly higher for the objects with lower complexity (F(1, 22) = 22.9, p < .0001, η^2^_p_ = 0.51). While there was a significant main effect of stimulus presentation type (F(4, 22) = 4.2, p < .02, η^2^_p_ = 0.44), the variations in presentation type only differentially affected the participants’ performance for objects with lower complexity (i.e., there was a presentation type x complexity interaction, F(4, 22) = 3.2, p < .04, η^2^_p_ = 0.37). There was no significant variation in performance across the various presentation types for the stimulus objects with higher complexity (note that the error bars for the high complexity conditions in Fig 8 overlap substantially). Interestingly, the effects of motion (evident in the results shown in Fig 7) did not vary by complexity (i.e., there was no motion x complexity interaction, F(1, 22) = 0.04, p = .85): the presence of motion or active haptic exploration was equally beneficial for the low- and high-complexity stimulus objects.

An overall comparison between the results of Experiments 1 and 2 is shown in Fig 9. It is clearly evident that while the presence of motion/active haptic manipulation resulted in significant improvements in performance for both object types (F(1, 47) = 74.4, p < .000001, η^2^_p_ = 0.61) that the enhancement obtained for the bell pepper stimuli was much higher than that that occurred for the glavens (i.e., there was a motion x object type interaction, F(1, 47) = 13.4, p < .001, η^2^_p_ = 0.22). There was also a significant main effect of object type, such that overall, the participants’ shape-matching performance was higher for the bell pepper objects (F(1, 47) = 6.6, p < .02, η^2^_p_ = 0.12).

## General Discussion

**Fig 10:**
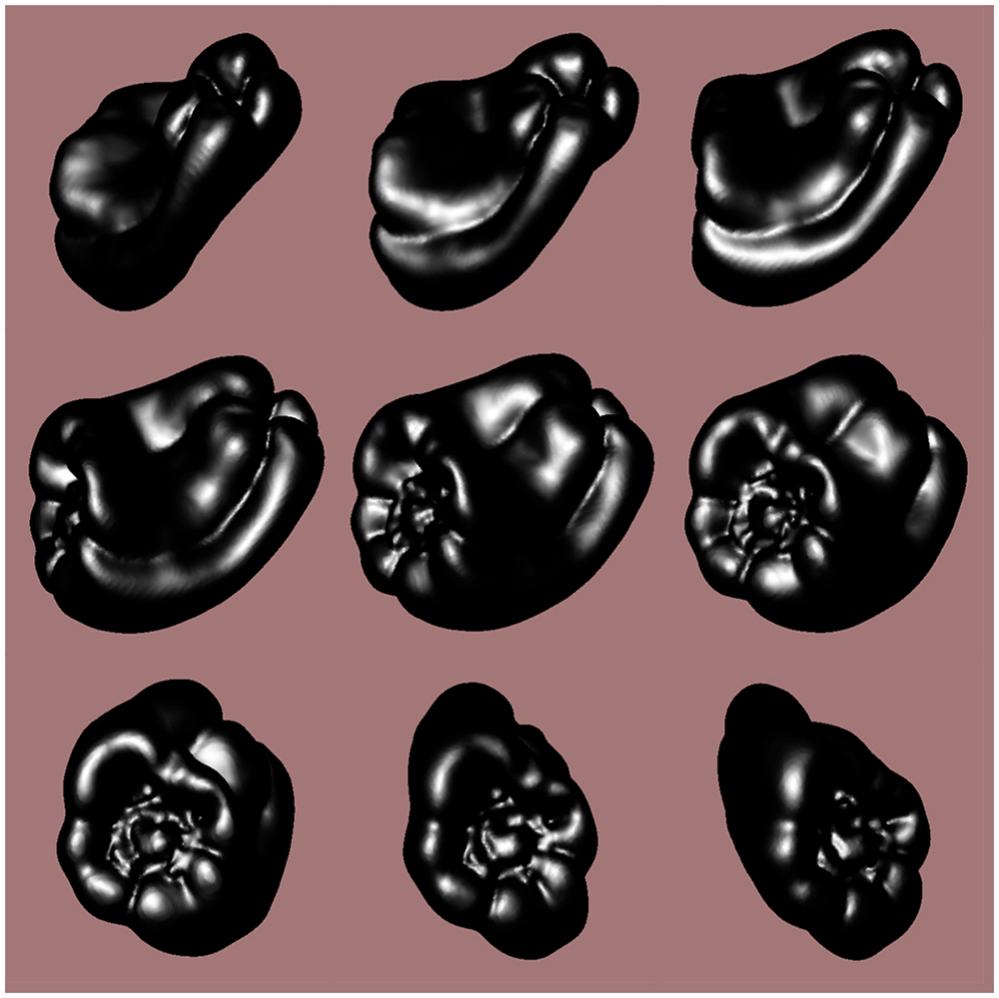
A sequence of views of a shiny solid object (bell pepper 11) rotating in depth; the views progress from the upper-left (first view) to the bottom-right (last view). The object in each view has been rotated by 20 degrees from its orientation in the previous view. It is important to note that the shiny specular highlights deform quite radically over time in response to the object rotation in depth relative to the environmental light source. Note also that the object’s outer boundary contour also deforms in a complicated manner over time. Both types of motion (specular highlight and boundary contour deformation) differ qualitatively from the optical motions of surface texture elements (when they exist).

In both Experiments 1 (replicas of bell peppers) and 2 (glavens), significant improvement in visual object identification occurred for the objects defined by texture (condition VT+BC in Figs 4 and 7) when motion, rotation in depth, was present. In our experiments, the volumetric texture condition is the “control condition” and the obtained improvement in performance (relative to the performance that occurred for the static condition) represents the "conventional" kinetic depth effect [3–9]. In our experiments, the participants’ performance improved (for the volumetric texture condition) by 30 and 114 percent for the glavens and bell peppers, respectively. It is well known [19, 22–23, 38–39] that specular highlights contain useful perceptual information about solid object shape. Our current results now demonstrate that the magnitude of the kinetic depth effect is as strong for stimulus objects defined only by specular highlights as for objects that are defined by surface texture: the participants’ object identification performance for the stimulus displays containing specular highlights (condition SH in Figs 4 and 7) improved with motion by 108.7 and 185.4 percent for the glavens and bell peppers, respectively. Little is known at present about how human observers actually extract information about 3-D object shape from optical patterns containing specular highlights. We do know that specular highlights generally cling to surface areas that are highly curved [19, 40] (e.g., see Fig 10). Unfortunately, computational models have not yet been developed that can successfully extract complete 3-D structure from specular highlights, probably because the optical deformations of specular highlights behave quite differently from the projected motions of surface texture [41–42] (e.g., see the apparent motion sequence depicted in Fig 10).

A comparison of Figs 4 and 7 show that dynamic (i.e., moving) boundary contours, all by themselves (condition BC), were perceptually quite informative in the current study and enabled accurate object identification for the naturally-shaped bell peppers, but not for the glavens. The optical deformation of boundary contours that accompanies object motion also differs fundamentally from the projected motions of surface texture elements [18, 21, 41–42] and successful extraction of 3-D object shape must occur via mechanisms that operate differently from conventional models of structure-from-motion (for reviews of this issue, see references [18, 21] & [43]). As a solid object rotates in depth, local regions of its outer boundary change both quantitatively and qualitatively as the rotation brings new 3-D structure to the projected boundary. Catastrophes in the boundary occur, for example, when a previously visible boundary feature (i.e., a particular convexity or concavity) disappears, or when a new boundary feature (a particular convexity or concavity) suddenly emerges that did not previously exist. Boundary catastrophes like these necessarily specify local 3-D shape [44–45]—it is possible to obtain information about an entire object’s 3-D shape from such boundary events if, like our participants, one is allowed to watch the entire rotation of the object. There is, therefore, no more optical ambiguity to specify shape for one class of objects (e.g., bell peppers) over another (e.g., glavens). In our experiments, however, the participants were much better able to utilize boundary contour deformation to identify bell peppers than glavens (our participants’ shape identification performance was approximately 50 percent correct for the bell peppers, but was only 30 percent correct for the glavens). This empirical difference is interesting, because it suggests that some objects produce boundary contour changes that are somehow perceptually more informative than others; further investigation of this issue is definitely warranted.

The results of Experiment 2 demonstrate that variations in object complexity are perceptually significant. When the participants’ performance (Fig 8) was examined separately for the lower and higher complexity glavens, it is clear that except for the specular highlights only condition, the participants’ object identification ability was generally quite good for the lower-complexity glavens. Apparently, too many (i.e., too high a density of) surface undulations makes it difficult for people to identify objects no matter how object shape is defined (vision, haptics, texture, specular highlights, boundary contours, etc). Interestingly, while people consider highly complex solid objects to be visually attractive and aesthetically pleasing [46], our current results demonstrate that increases in complexity can produce deteriorations in the ability to perceive shape (the average visually obtained performance for the high-complexity glavens in Experiment 2 was only 27.1 percent correct).

Finally, our current results demonstrate that the kinetic depth effect for haptics is quantitatively as strong as that obtained by vision (see Figs 4 & 7). While the overall visual object identification performance in the motion conditions was 30 percent higher than that obtained for the static conditions for the glavens (Fig 7), the corresponding increase in performance for active haptic exploration was 56 percent. For the naturally-shaped bell peppers, the increases in performance due to visual object motion and active haptic exploration were 98.9 and 76.7 percent, respectively. While we have known for decades that active haptic manipulation leads to better shape perception than more passive tactile/cutaneous stimulation [24, 28, 33, 47–49], it is very interesting that the magnitude of the haptic improvement in performance with motion (i.e., active object manipulation) is about as large as that that occurs for vision. This similarity in outcome for the modalities of vision and touch is striking (e.g., compare conditions H & VT+BC within both Figs 4 and 7) and reinforces recent findings [28, 50–52] that these modalities are in many ways functionally equivalent, despite the obvious differences in the information that is actually being detected (spatiotemporal optical patterns of light versus dynamic pressures applied to the skin & skin/tissue deformation) as well as the peripheral neurophysiology itself (e.g., the rod and cone photoreceptors within the retina of the eye versus mechanoreceptors within the skin of our fingers, etc.).

## Conclusions

Specular highlights and occlusion boundary contours are effective sources of visual information about solid object shape; deforming (moving) highlights and boundary contours enhance object identification as much as the enhancement that occurs with moving objects that possess surface texture. In addition, active haptic exploration produces as much of an enhancement in performance as visual object motion.

## Supporting Information

S1 Dataset Individual participant results for Experiment 1. (XLS)

S2 Dataset Individual participant results for Experiment 2 as a function of motion and presentation type. (XLS)

S3 Dataset Individual participant results for Experiment 2 as functions of motion, presentation type, and stimulus complexity. (XLS)
